# Trans health care from a depathologization and human rights perspective

**DOI:** 10.1186/s40985-020-0118-y

**Published:** 2020-02-19

**Authors:** Amets Suess Schwend

**Affiliations:** Research Group “Others. Feminist Perspectives in Social Research” (SEJ-430), University of Granada, Granada, Spain

**Keywords:** Depathologization, Human rights, Trans activism, Trans health care, Human rights in patient care, Research methodology and ethics

## Abstract

Trans people are exposed to multiple human right violations in clinical practice and research. From 1975 on, gender transition processes have been classified as a mental disorder in diagnostic classification manuals, a classification that was removed recently from ICD, International Classification of Diseases, and continues in DSM, Diagnostic and Statistical Manual of Mental Disorders. Trans people in different world regions are forced to accept psychiatric diagnoses and assessment in order to get access to trans health care, subject to reparative therapies and exposed to transphobic institutional and social discrimination and violence. In many countries, gender identity laws include medical requirements, such as psychiatric diagnosis, hormone treatment, genital surgery, or sterilization. In the scientific literature, a frequent pathologization of trans experiences can be identified, by means of pathologizing conceptualizations, terminologies, visual representations, and practices, as well as ethnocentric biases.

Trans activism and scholarship have questioned widely the pathologization of trans people in clinical practice and research. Over the last decade, an international trans depathologization movement emerged, demanding, among other claims, the removal of the diagnostic classification of transexuality as a mental disorder, as well as changes in the health care and legal context.

International and regional bodies built up a human rights framework related to sexual, gender and bodily diversity that constitute a relevant reference point for trans depathologization activism. The Yogyakarta Principles, published in 2007 and extended in 2017 by means of the Yogyakarta Principles plus 10, establish an application of international human rights law in relation to sexual orientation, gender expression, gender identity, and sex characteristics. International and regional human rights bodies included demands related to depathologization in their agenda.

More recently, advancements towards trans depathologization can be observed in the diagnostic classifications, as well as in the health care and legal context. At the same time, trans people continue being exposed to pathologization and transphobic violence.

The Human Rights in Patient Care (HRPC) framework offers a human right-based approach on health care practices. The paper aims at analyzing the shared human rights focus and potential alliances between the trans depathologization perspective and the HRPC framework.

## Background

All over the world, trans people^1^ are exposed to human rights violations, including social and labor discrimination, criminalization, pathologization, exposure to transphobic violence, and homicides [1–14]. Trans authors and allies observe an interrelation between these human rights violations and the contemporary Western medical model of transexuality that psychopathologizes gender expressions and identities which differ from the social expectations related to the sex assigned at birth [9, 11, 13–25]. This medical model, raised in the twentieth century, replaces partially and coexists with previous conceptualizations of gender transition as a sin or crime [15, 23] and is questioned by a more recent conceptualization of free gender expression and identity as a human right [26–34].

Gender transition processes continue being classified as mental disorders in the DSM-5, Diagnostic and Statistical Manual of Mental Disorders, 5th edition [35], published by the American Psychiatric Association in 2013. In the ICD-10, International Statistical Classification of Diseases and Related Health Problems, 10th edition, launched by WHO, World Health Organization in 1990, trans-related codes were placed in the chapter “Mental and Behavourial Disorders” [36]. On 25 May 2019, the World Health Assembly approved ICD-11 [37], published online by WHO in June 2018 [38]. In ICD-11, all trans-related diagnostic codes were removed from the chapter “Mental and Behavioural Disorders,” and the code “Gender incongruence” was included in a new chapter “Conditions related to sexual health” [38]. In spite of this recent advancements, in different world regions, trans people continue receiving psychiatric diagnoses, are forced to reparative therapies aimed at modifying their gender expressions or identities, or have to undergo psychiatric assessment processes based on binary and heteronormative assumptions in order to get access to hormone treatment or trans-related surgeries [1, 2]. In many countries, Gender Identity Laws establish diagnosis, hormone treatment, genital surgery, sterilization, and/or divorce as requirements for legal gender recognition [4, 5, 26, 39–41]. Furthermore, trans people continue being subjected to discrimination and transphobic violence [1–4, 6–8]. An emerging field of trans studies and allies identify a frequent pathologization of trans experiences in research, by means of discriminatory conceptualizations, terminologies and visual representations, the promotion of clinical practices that do not fulfill human rights standards, and ethnocentric biases [42–53]. They observe a frequent lack of ethical practices in research processes with trans people, such as an absence of informed consent, violation of confidentiality, and lack of opportunities for active participation in research processes [42–44, 49, 51]. Furthermore, they criticize the exclusion of trans people from knowledge production processes, with a frequent reduction to a “testimony” role, without recognizing the theoretical contribution of trans scholarship [47].

Over the last decade, international trans depathologization activism and scholarship emerged that denounce current diagnostic classifications, pathologizing clinical practices, legal frameworks, and research practices and propose alternative frameworks [26–34, 54–74], preceded and accompanied by critical theoretical reflections contributed over the last decades [11, 13–25].

These discourses identify the human rights framework as one of the most relevant foundations of the depathologization perspective. Taking into account this relevance, this paper aims at analyzing the relationship between trans depathologization discourses and the Human Rights in Patient Care (HRPC) framework [75, 76].

HRPC refers to “the application of human rights principles to the context of patient care” (p. 7) [75]. HRPC builds up on international human rights law, as established in several international covenants, conventions, and charters, supporting rights that are relevant in the health care context, such as the right to liberty and security of person, the right to privacy and confidentiality, the right to information, the right to bodily integrity, the right to life, the right to the highest attainable standard of health, the right to freedom from torture and cruel, inhuman and degrading treatment, the right to participation in public policy, the right to non-discrimination and equality, and the right to remedy [75]. HRPC establishes differences from a consumer-oriented patients’ rights approach and defines itself as complementary to bioethical perspectives [75, 76], focusing on “universal, legally recognized human rights principles, protecting both patients and providers” (p. 7) [75]. The HRPC framework can be related to the Human Rights-Based Approach to Health Care developed by WHO that “aims at realizing the right to health and other health-related human rights” (p. 1) [77].

Before presenting the depathologization and human rights perspective and analyzing its relationship with the HRPC framework, I would like to add some words on my own perspective and trajectory, according to the principles of self-reflexive research practice [30, 78, 79]. I work as a trans academic, activist and artist, and intersex ally on trans and intersex depathologization; human rights, sexual, gender, and bodily diversity; and research epistemology and ethics. In my PhD, I analyzed the trans depathologization perspective and its contribution to research epistemologies, methodologies, and ethics [30]. Over the last decade, I have participated in international networks and expert groups working on trans depathologization and human rights. In the artistic field, I use performance and other artistic techniques to reflect on trans depathologization and gender binarism. This background places me in a position of specific collective responsibility when writing in an academic context.

## The depathologization perspective

Pathologization can be understood as the conceptualization of bodily characteristics, habits, practices, living forms, gestures, people, and groups of people as mentally disordered, ill, abnormal, or malformed [28]. The demand for depathologization is based on the observation of multiple forms of pathologization of trans people in different social fields, including the family, social, educational, academic, labor, clinical, and legal context [1–14].

Over the last decade, an international trans depathologization activism emerged in the scope of the parallel review of the diagnostic manuals DSM and ICD [26–34, 47, 54–74, 80, 82]. In October 2007, the first parallel coordinated demonstrations were organized in various European cities, organized by a network of local trans groups (International Network for Trans Depathologization) [30]. From 2009 on, each month of October the International Day of Action for Trans Depathologization has been celebrated, convened by STP, International Campaign Stop Trans Pathologization [28, 30, 33, 80]. Around 250 groups and networks from different world regions participated between October 2009 and October 2017 in more than 795 activities in 183 different cities within the International Day of Action for Trans Depathologization [80]. Trans activist groups and networks worldwide published reports, declarations, and press releases demanding trans depathologization [30, 80].^2^ These local groups in different world regions show a great culturally and geopolitically diversity regarding organization forms and priorities [30, 70]. In order to avoid ethnocentric imposition, STP invited local groups to add their regionally specific demands to the shared objectives for trans depathologization [30, 70]. In the last decade, international and regional networks became stronger, establishing forms of collaboration and lobbying activities in international and regional human rights bodies [30, 81, 82].

The most relevant demands for trans depathologization activism include the removal of the diagnostic classification of gender transition processes as a mental disorder from the DSM and the ICD, public coverage of trans health care, as well as a change in the trans health care model, from a psychiatric assessment process towards an informed decision-making approach. Furthermore, trans depathologization activism claims legal gender recognition without medical, civil status, age or nationality requirements, depathologization of gender diversity in childhood, protection from discrimination and transphobic violence, and depathologization of research practices [28, 30, 33, 80]. Over the last decade, the demand for a removal of the diagnostic classification of transexuality as a mental disorder has received the support of professional associations [30], as well as European human rights bodies [83–92].

In the following paragraphs, I will summarize relevant principles and demands of trans depathologization activism and scholarship, as well as recent achievements in the health care and legal context, relating them with the human rights perspective established in the Yogyakarta Principles and Yogyakarta Principles plus 10 [93, 94] and the HRPC framework [75, 76].

## Principles

### Human rights framework

A reciprocal relationship can be observed between the international human rights framework and trans depathologization activism and scholarship [30]. The human rights framework can be identified as a relevant reference for trans depathologization [28, 30, 33]. At the same time, European human rights bodies incorporated trans depathologization perspectives in their agenda and strategic documents [83–92].

Over the last decade, an application of fundamental human rights principles to sexual, gender, and bodily diversity can be observed. In 2006, an international expert group developed the *Yogyakarta Principles, Principles on the application of International Human Rights Law in Relation to Sexual Orientation and Gender Identity* [93], published and presented at the UN Human Rights Council in 2007. The Yogyakarta Principles are a relevant reference document for international depathologization activism and scholarship. In 2017, the Yogyakarta Principles plus 10 were published, with additional principles that refer to new topics and priorities raised over the last decade, including aspects related to the human rights of intersex people [94]. In 2011, the UN passed the first resolution on non-discrimination on grounds of sexual orientation and gender identity [95]. From this moment on, UN agencies and regional human rights bodies released strategic documents related to the protection from discrimination on grounds of sexual orientation, gender expression/identity, and sex characteristics [83–92, 96–99]. Furthermore, in 2016, the mandate of an International UN Expert on Sexual Orientation and Gender Identity was established [100], considered as an opportunity for the defense of trans rights worldwide [101]. Several authors analyzed arguments present in the international human rights law in order to defend the right to depathologization [29, 32].

Over the last decade, European human rights bodies included demands of trans depathologization activism in their agenda, among them, the demand for a removal of transexuality as a mental disorder from ICD, public coverage of trans health care, legal gender recognition without medical requirements, and abolition of the diagnostic code “Gender incongruence of childhood” [83–92].

The relevance of the human rights framework for trans depathologization activism and scholarship establishes a direct relationship with the HRPC approach that “refers to the theoretical and practical application of general human rights principles to the patient care context, particularly to interactions between patients and providers” (p. 8) [75]. Furthermore, the HRPC framework establishes: “Complementary to—but distinct from—bioethics, human rights in patient care carry legal force and can be applied through judicial action” (p. 7) [75]. While a patients’ rights approach focuses on individual rights, the HRPC framework include a collective, public health perspective [77]. Taking into account this shared human rights framework, HRPC can be identified as a useful model for a trans health care practice based on a depathologization perspective, and the depathologization perspective can inform the HRPC approach, contributing additional aspects and priorities.

### Psychiatrization, discrimination, and transphobic violence

Trans depathologization activism and scholarship emerged from the observation of an interrelation between the conceptualization and diagnostic classification of gender transition as a mental disorder and the situation of discrimination, stigmatization, social exclusion, and transphobic violence trans people are exposed to in different world regions, including both forms of physical and institutional violence [9, 13–25]. Therefore, trans depathologization activism demands the removal of the diagnostic classification of transexuality as a mental disorder from the DSM and ICD, as well as the recognition of gender diversity as a human right and expression of diversity, placing the problem not in the person, but in the transphobic attitudes of the social context [26–34, 47, 54–74, 80].

From a transcultural perspective, trans-identified and allied authors highlight the absence of a conceptualization of gender transition processes as a disorder or illness in some non-Western cultures that recognize and celebrate gender diversity, assigning them specific cultural meanings [53, 101, 102]. At the same time, they highlight the importance of avoiding a romanticizing and ethnocentric view on the recognition of gender diversity in non-Western cultures [53]. Furthermore, they associate the demand of removing the diagnostic classification of transexuality as a mental disorder from DSM and ICD to a broader questioning of a psychiatrization of social phenomena, discrimination of mental health problems, and human rights violations in mental health care, establishing an alliance with the movement of (ex)users and survivors of psychiatry, as well as a relation to social anthropology, transcultural psychiatry, and antipsychiatry [30].

Furthermore, trans authors and allies refer to the colonialist character of an exportation of the Western medical model to other cultures, linking depathologization and decolonialization [46].

The demand of trans de-psychopathologization and questioning of stigmatization and human rights violations in mental health can be related to the right to protection from medical abuse and the right to freedom from torture and cruel, inhuman, and degrading treatment established in the Yogyakarta Principles (Principles 10 and 18) [93], as well as in several international human rights treaties [83–92, 95–99]. These principles are also relevant guiding principles for the HRPC framework: “A vast and severe range of human rights violations occur in the patient care context that violate rights in addition to the right to health, including many civil and political rights. In response to growing concern about this abuse in many parts of the world, the phrase and concept ‘human rights in patient care’ has recently grown in usage as a framework for monitoring, documenting, and analyzing abuses in patient care settings, and in some cases, holding governments and other parties accountable” (p. 7) [75]. The decolonialization perspective is not explicitly mentioned in the HRPC framework, but could be incorporated in HRPC practice. When applying the HRPC framework to trans health care in general, and particularly in the Global South and East, in migration, and intercultural contexts, the consideration of the link between depathologization and decolonialization becomes especially relevant.

### Gender non-binarism

Gender non-binarism can be identified as another relevant principle of the trans depathologization perspective [28, 30, 33, 65, 69] and previous reflections [17–19, 23, 24]. In opposition to a binary and heteronormative conceptualization of transexuality established in the diagnostic classifications, standards of care and gender identity laws, trans depathologization activism highlights the diversity of gender expressions, trajectories and identities, including non-binary and fluid options, as well as the diversity of trans people’s sexualities, challenging the clinical assumption that all trans people are heterosexual [30].

Recognizing the legitimacy of the desire for bodily modification, trans authors and allies question the presupposition of its compulsory character in the medical model [17–19, 22–25, 28, 30, 33, 65, 69, 72]. They identify the association of transexuality with bodily modification as a result of a binary and medicalized Western society that imposes a normative conceptualization over the sexed body, without taking into account the diversity of trans people's bodily trajectories and health care needs [46]. In this sense, trans depathologization activism and scholarship support a model of gender diversity in which different gender expressions, trajectories, and identities have the same recognition and human rights protection, including binary and non-binary options, with and without bodily modification [30].

These reflections are part of a broader discussion on human rights violations on grounds of gender and bodily diversity, gender binarism, and hetero-, cis-, and endonormativity contributed by queer theory [103–105], trans [1–34, 46, 47, 54–74] and intersex studies [106–108]. Queer studies question gender binarism and normativity, creating new conceptualizations for gender diversity and nonconformity [103–105]. Trans studies establish a relationship between gender binarism and dynamics of discrimination, pathologization, and transphobia [1–34, 46, 47, 54–74]. Intersex studies identify gender binarism as one of the grounds of a medical model of early genital surgery in intersex children, condemned as a human rights violation by the intersex movement, the UN and regional human rights bodies [106–110].

In the Yogyakarta Principles [93], Principle 18—Protection from Medical Abuses establishes that “States shall (...) A. Take all necessary legislative, administrative and other measures to ensure full protection against harmful medical practices based on sexual orientation or gender identity, including on the basis of stereotypes, whether derived from culture or otherwise, regarding conduct, physical appearance or perceived gender norms” (23). In the Yogyakarta Principles plus 10 [94], Principle 32—The Right to Bodily and Mental Integrity indicates that “States shall: (...) C. Take measures to address stigma, discrimination and stereotypes based on sex and gender, and combat the use of such stereotypes, as well as marriage prospects and other social, religious and cultural rationales, to justify modifications to sex characteristics, including of children” (10).

The HRPC framework does not refer explicitly to gender non-binarism, but it includes a mention of the right to bodily integrity and freedom from torture, cruel, inhuman, and degrading treatment [75]: “The concept of human rights in patient care provides a framework for addressing abuses in health settings and holding governments accountable for them. (...) Includes key patient rights to liberty and security of the person; privacy; information; bodily integrity; life; highest attainable standard of health; freedom from torture, cruel, inhuman, and degrading treatment; participation in public policy; non-discrimination and equality” (p. 16-17). The awareness of gender non-binarism can be identified as a specific contribution of the depathologization perspective to the HRPC framework, especially, but not only relevant when applied to trans health care.

## Demands and recent developments

Apart from these main principles, the international trans depathologization activism expressed several demands and developed proposals and suggestions responding to recent developments.

### Removal of the diagnostic classification as mental disorder and state-funded coverage of trans health care

One of the main demands of the international trans depathologization activism is the removal of the diagnostic classification of gender transition as a mental disorder from DSM and ICD [26–34, 46, 47, 54–74, 80, 82]. Trans people from different world regions expressed their concerns regarding a loss of access and state-funded coverage of trans health care, or an increased difficulty for achieving it, in the case of a complete removal of trans-related diagnostic codes. As another fear, they mentioned the loss of opportunities for legal gender recognition in those countries in which gender identity laws require diagnosis [111, 112].

Responding to these concerns, the international trans depathologization activism developed different strategies, contributing (1) an argumentation framework based in the right to health, the right to health care, and the right to legal personality as established in international human rights treaties, as well as in the Yogyakarta Principles; (2) the consideration of contextually specific priorities, taking into account the variety of health care systems and legal frameworks in force worldwide; and (3) the recommendation of strategies in the short term (health care access and legal gender recognition) and long term (in-depth change of the health care systems and legal frameworks) [28–30, 32, 33, 63, 66, 68–70, 72–74, 80]. Furthermore, STP added the demand of state-funded coverage of trans health care to its main demands [113], and an international expert group coordinated by GATE, Global Action for Trans Equality elaborated reports with suggestions for the ICD revision process taking into account the relevance of health care access [114, 115].

Regarding DSM, trans depathologization activism maintained the demand of a complete removal of trans-related diagnostic categories [26–28, 30, 31–33, 80]. In relation to ICD, taking into account its character as a classification not only of mental disorders or illnesses, but of all types of health processes that might require health care, the international trans depathologization activism proposed the removal of trans-related codes from the chapter “Mental and behavioural disorders.” At the same time, the inclusion of a non-pathologizing code in a different ICD chapter was suggested, as a health care process not related with disorders or illnesses [26, 28, 30, 33].

In 2013, the APA (American Psychiatric Association) published the DSM-5, in which the diagnostic category “Gender Identity Disorder” was substituted by “Gender Dysphoria”, and the category of “Fetishistic transvestism” by “Transvestic disorder” [35]. The international trans depathologization activism criticized the continued diagnostic classification of transexuality as a mental disorder, as well as the expansion of the category ‘Transvestic disorder’ by means of the ‘autogynophilia’ concept [28, 30, 31, 33, 80].

In the ICD revision process, all diagnostic codes related to gender expression/identity and sexual orientation were removed from the chapter “Mental and behavioural disorders” in ICD-11 [38]. A code “Gender incongruence” was included in the new chapter “Conditions related to sexual health,” with two subcodes “Gender incongruence in adolescence and adulthood” and “Gender incongruence of childhood” [38].

Trans depathologization activism considered this change as an important advancement [80, 82]. At the same time, international and regional trans depathologization networks questioned the continued diagnostic classification of gender diversity in childhood [1, 2, 26–28, 30, 33, 54–62, 80, 82] and criticized the psychopathologizing connotations of the term “Gender incongruence,” proposing alternative terminologies, such as “Health care related to gender transition” [30].

The demand of a removal of the diagnostic classification as a mental disorder can be related to the Yogyakarta Principles, especially Principle 2—The Rights to Equality and Non-Discrimination and Principle 18—Protection from Medical Abuses [93]. The demand of state-funded trans health care of the highest attainable quality can be related to Principle 13—The Right to Social Security and Other Social Protection Measures and Principle 17—The Right to the Highest Attainable Standard of Health [93]. These principles are also relevant aspects for the HRPC framework: “A particularly important (though not exclusive) source of international human rights law relevant to patient care is the right to the highest attainable standard of health found in Article 12 of the ICESCR” (p. 10) [93], opening a shared ground for advocacy on the right to health care.

### Trans health care models

In parallel to the DSM and ICD revision process, international trans depathologization activism [26–28, 30, 33, 69–72, 80] focused over the last years on a third document, the SOC, Standards of Care for Gender Identity Disorders, elaborated by HBIGDA, Henry Benjamin International Gender Dysphoria Association, now WPATH, World Professional Association for Transgender Health [116, 117]*.* From 1979 on, HBIDGA/WPATH has been publishing periodically new versions of the SOC, developed initially from and for the US context, and applied in different world regions [116, 117]*.*

Regarding the SOC-6, published in 2001 [116], trans depathologization activism and scholarship [30, 69–72] criticized the psychiatric assessment regulating the access to trans-related hormonal treatments and surgeries, the application of the diagnostic codes established in DSM and ICD, the assumption of a binary transition process and heterosexual orientation of trans people, and the requirement of the “real-life experience,” i.e., the requirement of living full time in the desired gender and contributing proofs of this process. Furthermore, they questioned the presupposition that all trans people wish to follow a “triadic therapy,” including real-life experience, hormone therapy, and surgery. As another critical aspect, they highlighted the exclusion of intersex people from trans health care. From different world regions, trans depathologization activist groups contributed proposals for a model of trans health care based on information, counseling, accompaniment, and informed decision making [26, 30].

In 2012, WPATH published the *SOC-7*, *Standards of Care for the Health of Transsexual, Transgender and Gender Nonconforming People* [117]. Trans depathologization activism [26, 30, 80] valued positively the recognition of gender transition processes as not pathological, the acknowledgment of a wide diversity of gender expressions, trajectories, and identities and differentiated situations regarding trans health care according to the cultural and geopolitical context, the intention of using a non-discriminatory language, and the explicit condemnation of so-called reparative therapies. At the same time, they questioned the continuation of a psychiatric assessment model, the requirement of a “12-month experience of living in an identity-congruent gender role” (p. 60) [117], as well as the use of pathologizing approach and language in the section on trans health care for intersex people.

Recently, changes in the trans health care models can be observed in some world regions, with informed decision-making models implemented in some countries and regions, among them in Community Trans Health Care Centers in the US [118, 119], as well as in the Public Health Systems of Argentina [120] and some Spanish regions [26, 27].

The demand for a trans health care model based on information, counseling, and informed decision making can be related to the Yogyakarta Principles, specifically Principle 18—freedom from Medical Abuses [93] and Principle—32, The Right to Bodily and Mental Integrity [94]. As mentioned before, the right to information, right to counseling, right to consent, right to free choice, and right to personalized treatment are also relevant for the HRPC framework [75, 76].

### Legal gender recognition

Legal gender recognition without medical requirements is another relevant demand for international trans depathologization activism [5, 7, 26–34, 39–41, 63–70, 72–74, 80].

Recent studies identify a lack of gender recognition laws in many countries worldwide [1–5, 7, 10, 30, 39–41]. In those countries that count on Gender Identity Laws, they note a frequent presence of medical requirements, among them diagnosis, hormone treatment, genital surgery, and sterilization. Furthermore, requirements related to civil status (single status or divorce) are observed, as well as restrictions regarding age (limitation to people over 18) or nationality (exclusion of residents from other nationalities) [1–5, 7, 10, 30, 39–41].

Trans depathologization activism demands legal gender recognition without medical requirements or those related to civil status, age or nationality, and trans activist groups from different world regions work on the introduction or modification of gender identity laws without pathologizing requirements in their specific contexts [7, 26–28, 30, 33, 39–41, 63, 64, 66–70, 72–74]. This demand has been supported by European human rights bodies [83–92]. As a future demand, the abolition of gender markers from birth certificates, identity cards, and passports is claimed [30].

Over the last few years, the international trans depathologization movement celebrated advancements regarding legal gender recognition [7, 26–28, 30, 33, 39–41, 63, 64, 66–70, 72–74].

In 2012, the Argentinian Gender Identity Law (Ley 26.743) was passed [120], allowing legal gender recognition without medical requirements, including children and adolescents, under specific protection measures, with reference to the Convention on the Rights of the Child [121]. Taking the Argentinian Gender Identity Law as a reference point, over the last few years, gender recognition laws without medical requirements have been approved in several countries, among them 2014 in Denmark, 2015 in Mexico City, Colombia, Ireland, and Malta, 2016 in Bolivia, France and Norway, and 2018 in Portugal, Costa Rica, Chile, and Uruguay [26–28, 30, 33, 39–41]. In other countries, gender identity laws in force have been modified [30]. Nevertheless, in some of the named countries the law requires a court procedure for the sex markers change or maintains the requirement of clinical assessment for children and adolescents, limiting thus full gender self-determination [26].

The Yogyakarta Principle 3 establishes the right to recognition before the law [93], and Principle 31 of the Yogyakarta Principles plus 10 refers to the right to legal recognition [94]. The HRPC framework does not mention explicitly this right [75, 76]. Nevertheless, when applied to trans health care, these rights achieve relevance, due to the tight relationship between diagnosis and legal recognition still established in many gender identity laws, and the health impact of a lack of legal and social gender recognition.

### Depathologization of gender diversity in childhood and adolescence

Over the last few years, the demand of depathologizing gender diversity in childhood and adolescence has achieved an increased relevance in trans depathologization activism [1, 2, 28, 30, 33, 54–62, 122–125], including the following demands: (1) removal of the diagnostic classification of gender diversity in childhood from DSM and ICD; (2) support to gender diversity in childhood and adolescence in the family, social, school, and health care context; and (3) legal gender recognition for children and adolescents.

Regarding the diagnostic classification of gender diversity in childhood, various international and regional activist networks published declarations demanding the removal of the diagnostic code “Gender incongruence of childhood” from ICD, and trans authors and allies contributed critical theoretical reflections on the diagnostic classification of gender diversity in childhood in the DSM and ICD [1, 2, 28, 30, 33, 54–61], preceded by critical reflections elaborated over the last decades [20, 21]. This demand also received the support of clinicians and researchers [62] and European bodies [90, 91].

Among the main arguments in favor of removing the diagnostic code, trans authors and allies highlight the lack of clinical utility, the Western character of a conceptualization of gender diversity in childhood as a problem that requires health care, the potential stigmatizing effect, and a contradiction between a removal of diagnostic codes related to sexual orientation and the maintenance of the Gender Incongruence of Childhood code [1, 2, 20, 21, 28, 30, 33, 54–62]. Furthermore, the critical discourses counter reasons contributed by the defenders of the diagnosis [126, 127], arguing that a specific diagnosis for gender diverse children is not necessary for covering psychological support, justifying access to puberty blockers, or promoting research and training [1, 2, 20, 21, 28, 30, 33, 54–62].

Trans depathologization activists and allied professionals defend the right of children and adolescents to free gender expression, including non-binary or fluid options [30, 56, 122–125]. They stress the need of supporting children and adolescents to express their gender in the family, social, educational, and health care context, by facilitating safe spaces for the exploration of different gender expressions and identities and protecting them from discriminatory and transphobic attitudes, without forcing them into a binary transition. In the health care context, they recommend the provision of support and accompaniment, avoiding a medicalization of gender diversity in pre-adolescent children [30, 56, 122–125]. Trans authors and allies also refer to the right of adolescents to access hormone blockers [30, 56]. At the same time, they express concerns about potential health and social risks [30, 56]. They recommend health professionals to facilitate gender diverse children, adolescents and their parents contacts with family associations that support gender diversity and gender diverse / trans youth groups [30, 56].

Regarding legal gender recognition, trans depathologization activist networks and authors stress the right of children and adolescents to change their gender markers [26, 30, 56]. They value positively the possibility of not inscribing the sex assignment at birth, as established in the *Gender Identity, Gender Expression and Sex Characteristics Act,* passed 2015 in Malta, as well as the option of several changes, as regulated in the Norwegian Gender Identity Law [26].

The Preamble of the Yogyakarta Principles [93] states that “in all actions concerning children the best interests of the child shall be a primary consideration and a child who is capable of forming personal views has the right to express those views freely, such views being given due weight in accordance with the age and maturity of the child” (9). Several principles established in the Yogyakarta Principles [93] and Yogyakarta Principles plus 10 [94] include a specific reference to children, such as the Principle 13—The Right to Social Security and to other Social Protection Measures; Principle 15—The Right to Adequate Housing; Principle 16—The Right to Education; Principle 18—Protection from Medical Abuses; Principle 24—The Right to Found a Family; and Principle 32—The Right to Bodily and Mental Integrity.

### Depathologization of research practices

Trans authors and allies review critically dynamics of pathologization and discrimination present in clinical and social research [13–16, 23, 30, 42–53]. Questioning an external pathologizing gaze, they demand a recognition of trans authors with a double academic-activist background and contribute suggestions for non-pathologizing research practices.

Responding to the observation of a frequent pathologizing language use at conferences, WPATH and *EPATH, European Professional Association for Transgender Health* established working groups to develop ethical principles for guaranteeing a non-pathologizing and non-discriminatory use of conceptualizations, terminologies and visual representations, and avoiding a promotion of clinical practices contrary to human rights standards at the WPATH and EPATH conferences [45].

Furthermore, trans authors and allies contributed ethical reflections for studies on trans issues [13, 14, 30, 42–53], proposals for reducing cisgenderism in research practices [50, 52], recommendations for including gender diversity beyond the binary in quantitative methodologies [128], and for using a non-pathologizing language in the media [129].

Several of the principles established in the Yogyakarta Principles [93] and Yogyakarta Principles plus 10 [94] can be applied to the research field, such as Principle 2—The Rights to Equality and Non-Discrimination; Principle 6—The Right to Privacy; Principle 18—Protection from Medical Abuses; Principle 19—The Right to Freedom of Opinion and Expression; Principle 21—The Right to Freedom of Thought, Conscience and Religion; Principle 25—The Right to Participate in Public Life; Principle 26—The Right to Participate in Cultural Life; Principle 27—The Right to Promote Human Rights; Principle 30—The Right to State Protection; Principle 32—The Right to Bodily and Mental Integrity; Principle 36—The Right to the Enjoyment of Human Rights in Relation to Information and Communication Technologies; and Principle 37—The Right to Truth.

The critical gaze on pathologizing and discriminatory language also includes a critical review of the term “patient” [26]. From a trans depathologization perspective, the term “health care user” is given preference [26], proposing a non-pathologizing language use [26, 30, 45, 50, 52] that could inform the HRPC framework, especially when applied to trans health care.

## Conclusions

The review of the main principles and demands of the theoretical-activist trans depathologization perspective shows the relevant role of the international human rights framework, as established in the Yogyakarta Principles and reaffirmed in recent strategic documents. A reciprocal influence between the depathologization perspective and human rights discourses can be observed. This strong human rights focus allows a direct connection between the trans depathologization perspective and the HRPC framework, for being based both on the international human rights framework, as established in the Universal Declaration of Human Rights, international human rights law and the Yogyakarta Principles [93, 94].

The discussion of the right to health, the right to bodily integrity and autonomy, and the right to participation in health policies can be identified as shared priorities. As specific perspectives, trans depathologization activism and scholarship contribute a focus on depathologization, gender non-binarism, decolonialization, children’s human rights and legal gender recognition, questioning pathologizing practices and language use in the clinical and research context, including the critique of the term “patient.”

For developing a new trans health care model based on a depathologization and human rights perspective, the collaboration between trans activists, scholars, and health professionals can be identified as a relevant strategy. The HRPC framework offers an interesting starting point for establishing clinical practices and knowledge production based on a human rights framework that can be complemented by the depathologization perspective. This collaboration is not only relevant for trans health care, but for a human rights-based health care in general.

## Data Availability

Not applicable

## Abbreviations

APA: American Psychiatric Association
APTN: Asia Pacific Transgender Network
DSM-5: Diagnostic and Statistical Manual of Mental Disorders, 5th Edition
DSM-IV-TR: Diagnostic and Statistical Manual of Mental Disorders, 4th Edition, Text Revision
EPATH: European Professional Association for Transgender Health
GATE: Global Action for Trans Equality
HBIGDA: Henry Benjamin International Gender Dysphoria Association
HRPC: Human Rights in Patient Care
ICD-10: International Statistical Classification of Diseases and Other Related Health Problems, 10th version
ICD-11: International Statistical Classification of Diseases and Other Related Health Problems, 11th version
ILGA: The International Lesbian, Gay, Bisexual, Trans and Intersex Association
SOC: Standards of Care (published by HBIGDA/WPATH)
STP: International Campaign Stop Trans Pathologization
WHO: World Health Organization
WPATH: World Professional Association for Transgender Health

## Footnotes

1 Within this text, the term ‘trans’ refers to a person “*who identifies with a different gender and/or expresses their gender identity differently from the gender that they were assigned at birth*” [33]. The term is used taking into account its Western character that may not coincide with forms of gender transition or transgression in other cultural contexts [53].

2 A review of the activist documents for trans depathologization published between 2007 – 2015 can be found in Suess Schwend [30]. For consulting more recent documents, please visit the websites of international and regional trans and LGBT networks (Akatahá; APTN, Asia Pacific Transgender Network; GATE, Global Action for Trans Equality; Gender DynamiX; ILGA Asia; ILGA-Europe; ILGA World; Iranti; STP, International Campaign Stop Trans Pathologization and TGEU, Transgender Europe).
